# EXPERTISE, DISAGREEMENT, and TRUST IN VACCINE SCIENCE AND POLICY. The importance of transparency in a world of experts

**DOI:** 10.33392/diam.1871

**Published:** 2025-02-27

## Abstract

We discuss the relationship between expertise, expert authority, and trust in the case of vaccine research and policy, with a particular focus on COVID-19 vaccines. We argue that expert authority is not merely an epistemic notion, but entails being trusted by the relevant public and is valuable if it is accompanied by expert trustworthiness. Trustworthiness requires, among other things, being transparent, acknowledging uncertainty and expert disagreement (e.g., around vaccines’ effectiveness and safety), being willing to revise views in response to new evidence, and being clear about the values that underpin expert recommendations. We explore how failure to acknowledge expert disagreement and uncertainty can undermine trust in vaccination and public health experts, using expert recommendations around COVID-19 vaccines as a case study.

## 1 Introduction

In September 2021, the Joint Committee on Vaccination and Immunisation (JCVI) advising the UK Government decided to not recommend COVID-19 vaccines for children aged 12-15. As they stated,

“There is evidence of an association between mRNA COVID-19 vaccines and myocarditis. […] There is considerable uncertainty regarding the magnitude of the potential harms. The margin of benefit, based primarily on a health perspective, is considered too small to support advice on a universal programme of vaccination of otherwise healthy 12 to 15-year-old children at this time” (JCVI 2021)

A few days later, the UK Government decided to authorize mRNA COVID-19 vaccines for 12–15-year-old children. Did the Government give up on “follow the science”, the principle that was said to have informed its pandemic policy until then? Not quite. First, science experts were divided on the issue. Some experts were recommending COVID-19 vaccines for 12–15-year-old children for their medical benefits, including e.g. officials at the US Centers for Disease Control and Prevention (Iacobucci 2021, CDC 2021). Some experts were not. Second, the JCVI’s recommendation only concerned the medical aspect of the issue, that is whether the risks of side-effects outweighed the individual benefits in terms of protection from the risks of COVID-19.

The Government’s decision was based on a broader range of considerations, such as the potential for disruptions in school attendance and mental health costs due to restrictions that vaccinating children could have averted^1^. Ultimately, the Government concluded that uncertainty around the medical benefits of vaccination was not so large as to prevent authorizing -- and indeed, strongly recommending -- child COVID-19 vaccination. This decision was political. It was based on a value judgment about what counted as ‘enough’ certainty in the level of safety and effectiveness of vaccines and on political choices regarding the conditions for the relaxation of harmful restrictive policies for children (e.g., school disruptions).

Some vaccine experts were taking a cautious approach and acknowledging uncertainty. Others were more confident about the COVID-19 vaccines’ overall net medical benefit to children. Yet other types of experts were considering the wide array of children’s interests (e.g., school attendance) outside of solely preventing COVID-19 infection.

This article is about the trustworthiness of experts in the context of vaccination. We assume that knowledge (e.g. regarding relevant scientific data) is necessary but not sufficient for expert authority. In the sphere of public policy especially, but arguably more generally, expertise and expert authority require public trust. We argue that in public policy, both trust and expert status can be undermined by failures of transparency in the acknowledgement of scientific uncertainty and expert disagreement about scientific knowledge. We defend these claims first, and then apply them both to vaccine research and policy, particularly in the context of recent debates regarding COVID-19 vaccines. In this paper, we are not aiming to solve the problem of whom we should trust in cases of expert disagreement. Instead, we argue that experts can improve their trustworthiness among general public, when they openly acknowledge two things. First, relevant uncertainties regarding knowledge claims. Second, that disagreement between experts can exist, due in part to uncertainty and in part to different value judgements.

## 2 Trust and Expertise: the Importance of Trustworthiness of Experts

The trust we are concerned with is a form of reliance on someone else, an expert, considered to possess knowledge or skills that are relevant to specific goals in which we have some stakes, - often (but not necessarily), skills and knowledge that we don’t possess (McLeod 2021). That is, we refer to trust in an epistemological sense. One can also trust someone for ethical reasons, for instance when you trust someone’s good intentions, or commitments to some ethical principle, or capacity for moral judgements. The two types are often related. On some views, trust in someone is always also trust in some moral skills, such as being honest, or caring about others, or having ‘goodwill’, (Jones 1996, McLeod 2021) or, we might add, someone’s professionalism. Even if one rejects such general accounts of trust, it is more difficult to reject the idea that trust in someone’s *expertise* specifically does involve a moral component. Having trust in someone’s expertise typically requires trusting their commitment to relevant ethical principles which are essential both for acquiring knowledge and for putting knowledge into practice. These include, for example, humility to acknowledge the limitations of their knowledge and the boundaries of their areas of expertise; being honest and transparent in acknowledging uncertainty and disagreement; or being committed to resolving conflicts of interest by prioritizing professional obligations over personal benefits. Guidelines for epidemiologists and public health policymakers often recognize the importance of trust and the key role played by ethical values in fostering public trust. For example, the American College of Epidemiology states in its Ethics Guidelines that “[t]rust is an expression of faith and confidence that epidemiologists will be fair, reliable, ethical, competent, and nonthreatening” (ACE 2000).

Because trust is a form of reliance on others’ skills, knowledge, and moral traits, trustors are dependent, for certain purposes, on those who are trusted. This puts trustors in a position of vulnerability, i.e., susceptible to being wronged or harmed by those who are trusted. Trust is therefore a double-edged sword. When we trust someone, it is because we think or hope this person is trust*worthy*, although that might not be the case. Experts need to be trusted not just because they are credible, that is, likely to be believed. They also need to be trusted because they are trust*worthy*, that is, deserving of trust (O’Neill 2020) - both trust in their knowledge and in the ethical approach to using that knowledge in practice.

In the healthcare context in particular, trust is linked to better healthcare service utilization, adherence to treatment, and self-reported health (Larson et al. 2018). Trust in society at large can facilitate effective implementation of public health interventions, perhaps particularly during public health emergencies.

As we suggest in the next section, the connection between trust and expertise is not only political and ethical - that is, it is not only about the value of trust in expert authority in liberal democracies. It is arguably also a conceptual relationship. We argue that trust is constitutive at least of a certain type of expertise. That is, expertise about matters that affect the interests of the general public or relevant portions of it. One cannot be that type of expert without being *trusted as* an expert. The very same notion of expertise and of expert authority would be undermined by the erosion of trust. Trustworthiness ensures that expertise is a useful, beneficial societal good.

One might think that that trustworthiness is based, among other things, on transparency regarding epistemic limitations in one’s knowledge as well as to value judgments involved in evaluations of evidence. As applied to epistemic conditions, the capacity to recognize uncertainty is itself part of what it means to possess knowledge in a certain domain. Transparency as an ethical requirement therefore arguably entails acknowledging ignorance, uncertainty, and expert disagreement, where relevant. The JCVI’s statement quoted earlier is a good example of how expertise is consistent with acknowledging uncertainty.

There are longstanding debates in philosophy of science regarding whether science is a purely empirical matter or the extent to which it is an activity laden with moral (and other) values (Kitcher 2001). When individuals are called on in their public role as experts and therefore their expertise affects the interests of the public, the latter aspect seems predominant: their evaluations of scientific evidence are typically grounded in value judgments regarding whether the level of available evidence or knowledge is *enough* to warrant a claim or recommendation - be it policy advice or advice on individual choices. This depends, for example, on whether we consider the risks of acting on uncertain data to be worth taking (Kitcher 2011, 35 and 148). Value judgements of this sort are unavoidable. Trustworthiness requires that experts are transparent about how values contribute to recommendations and to the judgment that a certain level of certainty is high *enough*, and avoid claiming that their judgements are “purely” scientific. In the words of Philip Kitcher, “[t]he deeper source of the current erosion of scientific authority consists in insisting on the value-freedom of Genuine Science, while attributing value-judgements to the scientists whose conclusions you want to deny” (Kitcher 2011, 40). We explore the issue of expert ‘authority’ below.

## 3 Expert and Expert Authority: a Matter of Trust

### 3.1 The authority of experts: a matter of trust

Do experts need to be always right about matters concerning their area of expertise to be legitimately considered experts and granted epistemic authority? The answer must be ‘no’. Surely, it is conceivable that experts are sometimes wrong, or even that experts are often wrong in certain circumstances. Again, this is a problem that epidemiology institutions sometimes acknowledge. For instance, IEA Guidelines of good practice state that “[e]pidemiologists should be wary of publishing poorly supported conclusions. History shows that many research results are wrong or not fully right, and epidemiology is no exception to this rule.” (IEA 2007)

Moreover, there is often uncertainty and/or disagreement among experts for reasons that do not have to do with epistemic failures. An outbreak of a novel virus or the development of a new vaccine technology are examples of such cases. While being wrong or being uncertain does not necessarily mean that someone ceases to be an expert, it might undermine one’s expert *authority* if not carefully managed. We define expert authority as the extent to which experts are trusted in their field of expertise to provide reliable information. We are not using the concept in a political sense, i.e. to describe the authority that experts are sometimes given in political decision making. We also want to keep it distinct from the notion of epistemic accuracy: the ‘authority’ we talk about often depends on perception of accuracy by a relevant audience, not simply on accuracy itself. Being wrong or uncertain about something might undermine this type of expert authority. An expert might make confident claims about the safety of a new vaccine even while data regarding rare adverse effects are still being collected. In the short term, this might sustain or increase expert authority and trust in vaccines; however, if a new vaccine turns out to be less safe than the expert initially claimed, this might undermine this type of expert authority (and trust in vaccines) in the long term.

Both expertise and expert authority are concepts that need to be unpacked. We start with the latter because, once authority is properly understood, it is easier to see the intimate connection between expertise, expert authority, and trust.

Moti Mizrahi claims that “the mere fact that an expert says that *p* does not make it significantly more likely that *p* is true” (Mizrahi 2013, p. 64.). Mizrahi bases this claim on several empirical considerations about the frequency with which experts in various fields have been proven wrong, and the magnitude of their mistakes. In this way, Mizrahi is not trying to question expertise itself. Of course, there are people who know more than others in a certain field and are more likely to get things right in the field. Instead, he wants to question – or at least to shed some healthy skepticism on - expert *authority*, understood as being trusted to provide reliable information in one’s field of expertise.

The term ‘significantly’ in Mizrahi’s claim points exactly to the issue of expert authority. Presumably, expert authority requires a *minimum* level of confidence in the reliability of expert claims, and not just *any* level that is higher than the mere chance of being right. After all, Mizrahi writes, “Would you trust a watch that gets the time right 55% of the time?”. One way to challenge Mizrahi’s skepticism about expert authority is to provide evidence that experts in any given field have been right significantly more often than, say, 55% of the time (Watson 2021, 72-76). We are not addressing this empirical issue here.

Instead, we want to point out that significant disagreement between experts would create a conceptual, and more challenging problem for our notion of expert authority. Suppose two otherwise similar experts (e.g. with similar track records of being correct, similar credentials, etc.) disagree on whether COVID-19 vaccination is, generally speaking, in healthy children’s best medical interests. This is essentially the situation we had when COVID-19 vaccines were authorized for use in adolescents and children. In such cases of disagreement, one might conclude that the two conflicting expert views would, so to speak, cancel each other out - neither group is ‘significantly’ more likely to be right than the other. People would still not know which experts to trust, that is, groups to which one should attribute authority.

An alternative view of expert authority relies on proxies for the probability of being right. Alvin Goldman (2001) suggests five possible criteria to decide whom to attribute epistemic authority to in case of expert disagreement. These are 1) the reasons each party can bring for and against the views at stake; 2) the extent to which other experts agree with either view (a kind of expert majority rule); 3) appraisal by ‘meta-experts’ (e.g., those providing credentials of expertise, such as academic degrees, professional accreditations, meta-researchers, and so on); 4) evidence of any conflict of interests or biases among experts; 5) experts’ past track records.

It is hard to see how any of these could be of much use in the scenario above to any parent who needs to decide which experts to trust. As for criterion 1, Goldman himself acknowledges that non-experts’ capacity to assess conflicting expert arguments is often limited. Parents might be interested in experts’ views on whether the risks of vaccines outweigh their medical benefits because, among other things, parents might not have the time and resources to master statistical methods or search for and assess evidence themselves. If they could use these tools to assess the veracity of experts’ claims, they might be eligible to be experts themselves. As for criteria 3 and 5, experts disagreeing on vaccines’ safety and efficacy may not have significantly different credentials or track records.

Depending on how the relevant expert community is defined, there might well be many experts agreeing with either position, as per criterion 2. However, “majority rules” is a fragile basis for expert authority, as majorities can be formed out of bad incentives. For example, Goldman says, some (putative) expert might belong to some “doctrinal community whose members devoutly and uncritically agree with the opinions of some single leader or leadership cabal” (p.98). A risk of creating such communities exists in domains characterized by heavy societal polarization (including vaccination policy). A polarized society or scientific community might well create non-financial incentives affecting experts’ claims on either side, although it may be difficult to assess the extent to which such incentives affect the veracity of experts’ claims (criterion 4). Non-financial incentives can be reputational, in terms of opportunities for career progression, or visibility or popularity among certain groups, and so on. The influence of such non-financial interests on professional conduct is widely acknowledged both in biomedical research (Saver 2012) and in health care (Wiersma et al. 2018). Vaccine experts are not immune from such dynamics. These incentives may bias expert evaluations of evidence, e.g., regarding the expected benefits and harms of novel interventions.

### 3.2 Expertise: a matter of trust

In this section, we explore the intimate connection between expertise and trust by pointing out some problems with alternative accounts of expertise that do not rely on it. Some critiques of expert authority, including Mizrahi’s one presented above, assume a certain conceptualization of expertise as a purely epistemic notion. They presuppose, more specifically, a “veritist account’ of expertise, whereby an expert is someone more likely to hold true beliefs or reliable beliefs within a certain domain - either more likely than most other people, or more likely than a certain threshold (Goldman 2018). Other kinds of epistemic bases for expertise could be provided, for example following certain procedures of scientific inquiry or certain methodologies for inferring conclusions.

However, we need to be careful when we select the relevant epistemic conditions for expertise. The idea that expertise is defined in terms of some special access to true propositions presupposes that expert status can only be determined from a standpoint where we already know the truth (Hardwig 1985). This is problematic for three reasons. First, such a standpoint might often be unachievable in principle (given intrinsic epistemic limits of human beings). Second, even if it were achievable, non-experts are unlikely to have access to all relevant facts, including those one would need to know in order to assess if someone making claims within that domain is an expert. Third, if non-experts had access to all relevant facts, not only might they be eligible to be experts themselves, but the notion of expertise would lose much of its practical purpose.

Indeed, it is the fact that someone we believe to be an expert makes a certain claim that provides good reason to believe that claim is true (Hardwig 1985), and not the other way round. In this sense, attribution of expertise is a matter of epistemic trust, because it implies giving credit to someone’s epistemic status without being ourselves in the position to assess the truth, or even the epistemic credentials, of their claims. It is true that sometimes we can test experts’ opinion *a posteriori*, via empirical verification of their predictions. For example, we could start vaccinating children and check if the outcomes are consistent with expert predictions and suspend our attribution of expertise until we find out which experts were right. However, we don’t typically wait to attribute expertise to individuals only *a posteriori*. Instead, we often rely on - and trust - their judgements before finding out if they are right. This intimate relationship between expertise and epistemic trust means that expertise is not an intrinsic property of an individual or a group thereof, which can be established simply through conceptual analysis and independently of the societal dynamics and relationships in which it is created. Instead, expert knowledge is any set of epistemic features that warrant *trusting* someone as an expert. Knowing the truth typically contributes to it, but it is not a necessary condition for it, for the simple reason that when we trust an expert, we cannot know if the expert claims are true. Thus, a conceptual analysis of whether someone is an expert is not separable from an assessment of whether a certain relationship of epistemic trust can be justified between that individual and the relevant community.

One option is to rely on peer-recognition as a reason to trust someone’s expertise, or on the institutions that confer certificates of expertise (such as academic degrees). Some take expertise to be the condition where one can contribute to domain-specific conversations with one’s peers, after one has learnt to master the language and methodologies specific to a certain domain (Collins 2014, Collins and Evans 2007). For example, experts in the JCVI and in the CDC disagreed with each other about whether the evidence available supported the claim that COVID-19 vaccines are in young adolescents’ best medical interests. Presumably, however, they shared a definition of ‘best medical interest” and criteria to establish such interests. On the domain-specific conversation view, the expertise of both groups is given by the fact that they can engage in this type of meaningful peer conversation, even though it might turn out that one of the two expert groups was incorrect. For example, experts who disagree could try to figure out possible respective mistakes, work together towards new positions, and so on. Individuals can engage in meaningful conversation, even in one that produces good results for society, while disagreeing about what is true.

This might well be a good proxy, or perhaps even a necessary condition for expertise. However, it is not sufficient. We would need reasons to trust the relevant communities of expert peers and/or to trust the institutions that confer certificates of expertise (such as academic degrees). This raises the question of what constitutes trust*worthy* expertise. At a minimum, there must also be some level of societal relevance of a certain field which warrants attribution of expertise - especially, as we suggested earlier, with expertise about matters that affect the interests of significant portions of the public. I could claim to be an expert on what I had for breakfast or someone else could claim to be an expert in alchemy, but these uses of the ‘expertise’ terminology are quite awkward. In neither case is the knowledge in question societally relevant. Unlike vaccines’ safety and effectiveness, what one person had for breakfast is typically uninteresting to anyone in society, and alchemy has effectively no current societal relevance (though of course there can be historical or anthropological relevance). Instead, experts are trusted because they are presumed to be better placed to assess how to meet needs and interests of individuals and society (Kitcher 2011). Fulfilling epistemic conditions defines expertise only to the extent that it is functional to a certain field being relevant in this sense to a relevant audience - in the case of expertise in public health, the relevant audience is the general public or significant portions of it. This suggests that expertise encompasses more aspects than just knowledge. It includes, in particular, the capacity to recognize relevant aspects of decision-making processes, such as how individuals’ and society’s interests will be affected - whether expertise is used to promote or infringe them (after all, expertise can also be used against societal interests); or distinctions between scientific claims and ethical-political values, so as to separate expert recommendations from political advocacy

**Figure 1:**
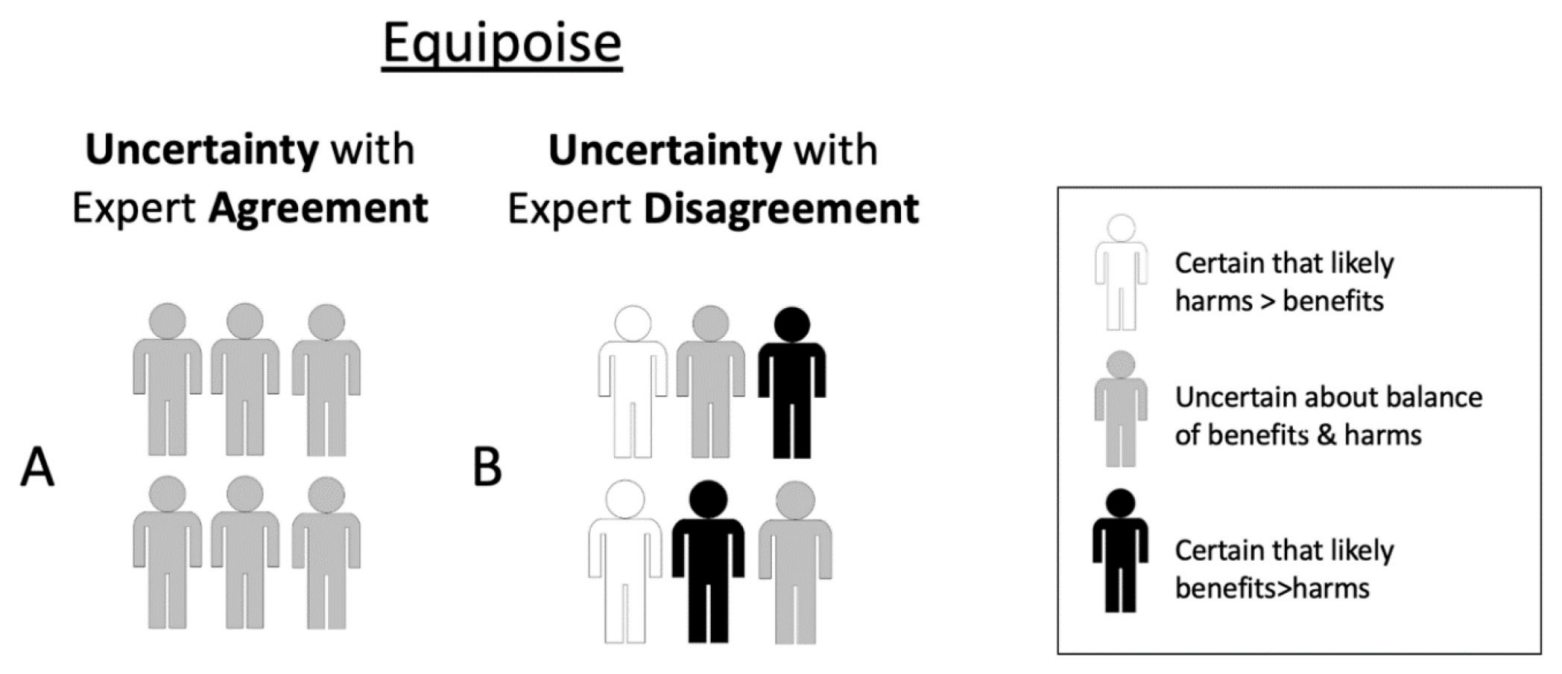
Equipoise

If this is correct, then it is impossible to separate expertise from being trusted as having expert authority. The epistemological problems that make it difficult, if not impossible, to determine expert authority based on access to true propositions would also make it difficult, if not impossible, to determine expertise on the same grounds. If expertise is a matter of trust, those with expert authority are those we have stronger, or sufficiently strong reasons to trust. These reasons can be of different sorts. We can *trust* their claims to be either true, or useful, or grounded in adequate training or qualifications, or based on certain methodologies of inquiry, or anything else that possesses societal relevance. After all, expertise is not only cognitive, but also performative: it consists in solving problems, giving advice, providing reasons for believing that something is true (Watson 2021, 170). This does not answer the question of whom we should trust in cases of expert disagreement. However, it makes expert authority independent of the answer to that question. It suggests that being right about things is neither sufficient nor necessary to give experts their authority. Topics covered by expertise, claims made by experts, and solutions experts propose need to be sufficiently relevant to a given audience. And experts with authority, that is, whom we have strong enough reasons to trust, can sometimes be uncertain (as individuals) and/or disagree with each other about current evidence (Figure 1). In both situations, experts are collectively uncertain even if some individual experts are certain. Both types of situations are consistent with (a significant degree of) so-called “equipoise”, that is, epistemic equilibrium between conflict claims resulting in significant uncertainty– an issue we will address in section 4 below.

### 3.3 Making experts trustworthy

There is an internal and external legitimization of experts’ claims and credentials (Moore 2017). Internal legitimization is the one established by criteria experts themselves in their own deliberative forums. However, as we have seen, that is not sufficient for expertise. Recognition of the relevance of certain fields matters as well. External legitimization is largely about public trust in the testimony of experts.

While it is unavoidable to ground many of our beliefs in epistemic dependence on experts, there might often be conditions that justify rejecting experts’ claims. These include, for instance, evidence that experts are refusing to acknowledge mistakes, or that they are subject to conflicts of interest including social pressure from others in the same field (Hardwig 1985, 342). Both can result in refusal to acknowledge uncertainty.

We often hear of a crisis of expertise, that is, decreasing levels of trust in experts (Watson 2021). However, it has been noted that overall levels of trust in experts do not drop, but simply shift among different domains of expertise. If we stop trusting certain experts, we will simply move that trust on to others (Watson 2021, 14). In the context of public health, pre-pandemic PEW Research suggested that public confidence in science and medicine in the US had been relatively stable, at about 40%, over the past three decades (Funk and Kennedy 2020). The same type of research during the pandemic has shown a sharp decline, dropping below 30% (Kennedy, Tyson, Funk 2021). Among other factors, perceived overconfidence in the medical field can be detrimental to trust (London 2021). Other fields, such as economics, have long accepted that overconfidence can undermine trust in experts and acknowledgment of limitations of experts’ knowledge can promote trust (Angner 2006).

A lack of trust in public health experts can be particularly serious insofar as public health experts need to enjoy what Matthew Bennet (2021) calls “recommendation trust”. In other words, trust that “I should do something because they have told me I should” (Bennett 2021, 248). Recommendation trust requires that recommendations are followed not out of fear of the consequences of disobedience, but because trust by itself provides enough reasons to follow them. In a liberal democracy, fear of consequences can be antithetical to trust as it can hinder contestation of experts. Freedom to contest expert claims may enhance trust by demanding transparent justification of recommendations. For example, vaccine critical activists may, by challenging mainstream narratives about the benefits of vaccines, create the demand for constantly improving the justifications for vaccination policies based on expert advice (Moore 2017, p. 55-57).

As we discuss below, debates in vaccine research and policy, particularly in the case of COVID-19 vaccines, exemplify issues around expertise and trust that we have presented so far. Questions about expert disagreement have arguably received greater attention in the context of research ethics (Smith 2021), especially within discussion of “equipoise” (see below). However, we suggest that policy surrounding COVID-19 vaccine research has failed to place enough weight on expert disagreement – for instance regarding the need to collect additional data on benefits and harms in specific groups before recommending or mandating vaccines (for relevant groups).

## 4 Equipoise in Research Ethics and Expert Disagreement

**Figure 2:**
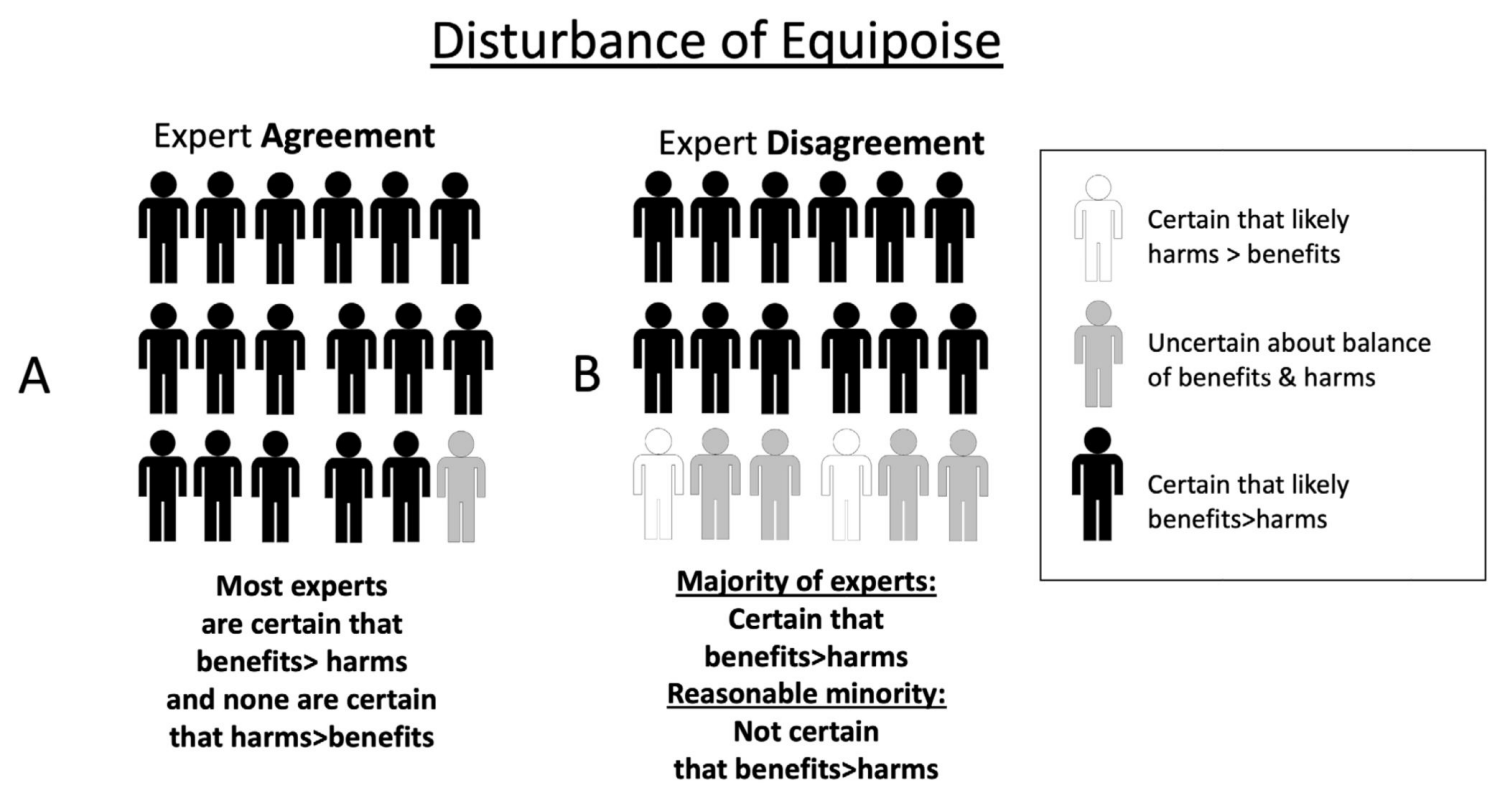
Disturbance of Equipoise with Expert Agreement versus Disagreement

In research ethics, questions about the degree of expert consensus have played a key role in debates about when it would be ethically acceptable to begin studies (especially randomized controlled trials) and/or when to terminate a study on the basis of data showing a clear net benefit or net harm associated with an experimental intervention. Initial theories of *equipoise* – the term commonly used in the field of research ethics - focused on the idea that an *individual* clinician would consider it ethical to enroll her patient in a clinical trial if the clinician was genuinely uncertain about whether an experimental treatment was better than standard care (Fried 1974). Later theories focus on equipoise of expert community opinions: a research study is considered ethical if, in the collective opinion of a *community* of well-informed experts, there is a sufficient degree of uncertainty and therefore equipoise between the expected balance of benefits and harms in the intervention and control arms in the study. In other words, the balance of expected benefits and harms of a new, experimental intervention versus an existing one that is used in the control arm is considered uncertain, given the disagreement among expert, in the given population under study (and for a potential participant as a representative of the study population) (London 2020). As illustrated in Figure 1, situations of community equipoise might sometimes occur when there is expert consensus (about uncertainty). They can also occur when there is expert disagreement, because some experts are certain that an experimental intervention is *superior* to an alternative (whether placebo or standard of care), some are uncertain, and some are certain that the experimental intervention is *inferior* to the alternative. Similarly, as shown in Figure 2, situations where equipoise is disturbed (i.e., where most experts are certain) can occur with widespread expert agreement (Figure 2A) or where there is some degree of expert disagreement (Figure 2B).

This raises questions about ethically acceptable approaches to situations of expert disagreement. A simple approach might be “majority rules”: once a majority of experts believe that evidence from research is sufficient to be confident that the expected benefits of an intervention outweigh its risks in a particular context, then research should stop, and the intervention should be implemented in policy. However, the history of science includes many examples illustrating that a minority of experts initially considered mistaken by the majority may sometimes later be proven right (Kitcher 1993). Thus, a “majority rules” approach to equipoise among experts would sometimes rule out ethically acceptable research where most experts *mistakenly* considered the research question settled (i.e., these experts were, taken together, certain that either the intervention or the control was overall superior in the population in question) but where a minority of experts *correctly* considered there to be a situation in which there is still significant uncertainty about the benefit-risk ratio of an intervention.

One way of accounting for disagreement in the context of judgements about community equipoise is to specify that equipoise persists where “at least a reasonable minority of experts […] would recommend that intervention for that individual” (London 2020) – i.e., the situation in Figure 2B. Of course, there might be different views about what a “reasonable” minority might be (and appropriate thresholds might vary in the context of different health problems, interventions, populations, etc.). However, this “reasonable minority” view at least provides a starting point to explain why research might sometimes be ethically acceptable even when a majority of experts consider that it would be unacceptable.

In the case of the rollout of mRNA COVID-19 vaccines, it was arguably ethically acceptable to collect additional data on the risks of myocarditis in young people because of the concerns of a minority of experts, even if a majority of experts considered such vaccines sufficiently safe for use in young people (Bardosh et al. 2022). Continuing to collect additional data might even be ethically required (rather than merely acceptable). This would be the case, for example, if there is reasonable disagreement that an *identifiable* group faces risks that outweigh benefit,s even if (there is widespread agreement that) benefits outweigh risks in the overall population to which the group belongs. Similarly, once COVID-19 vaccines were shown to be effective, it was arguably at least ethically acceptable to continue to collect data among recipients of placebos in initial trials, even if most experts thought that there was an ethical imperative to permit low risk placebo arm participants to access the vaccines despite not being in an otherwise eligible population group (Rid et al. 2021). However, it is important that this consideration is balanced with considerations around duties of care^2^.

In some cases, a minority of experts who remain uncertain (in equipoise) about the balance of benefits and harms of the vaccine in the population in question face a dilemma. If most experts (who believe the vaccine offers a net benefit over placebo) or members of the public become aware of the minority view, this minority group may face negative consequences as a result of holding such views (de Melo Martin and Intemann 2013). This might be especially likely to occur where (i) debates about vaccination in general become highly polarised, (ii) the vaccine in question is highly politicised, and/or (iii) the majority group seeks to ostracise and/or silence the minority group of experts. To resolve this dilemma, experts in a minority group may either falsify their preferences (i.e., publicly agreeing with the majority group while continuing to have private doubts about the majority position) (Kuran 1997) or publicly express their disagreement and accept any negative consequences.

Arguably, these patterns were observed during debates about COVID-19 vaccines. In some cases, they led to experts silencing themselves, being silenced by others, or being silenced in other ways (Bardosh et al. 2022). For example, the authorization of vaccine boosters for young healthy adults in the United States in the absence of efforts to collect more data resulted in the resignation of prominent vaccine regulatory experts at the US Food and Drug Administration^3^. This occurred in the context of wider debates about the merits of boosters in low-risk populations (Krause et al. 2021). Further, differences in expert opinion about COVID-19 vaccines between experts in different countries arguably revealed more uncertainty about a particular intervention at the global level than experts in one country might have been willing to admit (Bardosh et al. 2022).

The authorization of COVID-19 vaccines despite these debates conveys the idea that equipoise had been disturbed, i.e., that there is sufficient certainty among experts. By implication, further research would be considered unethical. Yet, a minority of experts considered that it was still uncertain that COVID-19 vaccines (in standard or booster schedules) offered net benefits for low-risk groups such as healthy adolescents, children, or those with immunity after previous infection (Pedgen et al. 2021).

### 4.1 Public health consensus and vaccine research

The goal of much vaccine research is to produce data to support new vaccines or refine the use of existing vaccines to promote net public health benefits. This goal raises questions about how much evidence is required before it would be appropriate to begin (or continue/discontinue) the roll out of a vaccine in public health practice. These questions arguably mirror those in research ethics, since as soon as vaccine trial data are considered to disturb equipoise in favor of the vaccine, at least some experts may recommend approval of the vaccine for public use (Cf. Figure 2B).

If one takes a “majority rules” view of consensus among experts, answers to such questions are straightforward. Once a reasonable majority of experts agree that the vaccine in question offers a superior balance of benefits over harms in a given population, the trial should be stopped, and the vaccine can be recommended accordingly. This is where expertise on vaccination science may overlap with expertise regarding public health policy. Within public health, the “majority rules” view about the ethical acceptability of implementing an intervention (supported by at least some research data) faces similar challenges to those in research ethics – especially where majority consensus conceals significant disagreement (Figure 2B). In public health practice, there are multiple potential sources of reasonable disagreement among experts, especially when interventions are novel, studies are few, or there are other epistemic limitations regarding the generalisability of existing results.

Debates about public health policy can be even more polarised than debates about research. Where at least some relevant research has been completed, some experts (sometimes most experts) may consider that the (net) benefits of the vaccine (or another intervention) have been proven beyond reasonable doubt. In such cases, it might be tempting to consider those who disagree as unreasonable or irrational. However, there will often be sources of reasonable epistemic and ethical disagreement.

First, experts might disagree about the extent to which one can have epistemic confidence based on existing (research) data that the intervention is superior to the control. This might be based on reasonable disagreement about the scientific design and conduct of relevant studies.

Second, disagreements about how to interpret existing data might sometimes be ethical disagreements about how much epistemic certainty should be required before implementing a new intervention in the real world. For example, “conservative” views would give more weight to the potential harms of low probability negative outcomes, such as vaccine side-effects that are not ruled out with high certainty by early data. “Early adopter” views would give more weight to the foregone benefits if implementation were slowed while conducting further research to collect more data (BMJ 1998). Both views are reasonable in different circumstances, but it is important to be clear about the extent to which different opinions reflect disagreement about science (or empirical and epistemic questions) versus disagreement about ethical and political values. Otherwise, the reasons for certain policies and the existence of reasonable debate may not be transparent to the public, which may undermine trust in public health experts. Experts’ policy recommendations are therefore always judgments about what level of evidence or knowledge is *enough*. This is a value judgment on the risks we are, or should be, prepared to take given relevant uncertainty. Reasonable disagreement about where the threshold for ‘enough’ lies can produce reasonable public health expert disagreement. This shows how public health expertise, and public health more generally, can never be only about empirical matters. Values underpinning expert recommendations might ideally be unpacked and discussed, but these values are not something on which vaccine experts have expertise. This is one reason why “follow the science” was never sufficient to justify pandemic policy, although it might have sounded reassuring to many and therefore there might have been good reasons to include it in public health messaging.

Third, experts might disagree about the degree of generalisability of knowledge generated by research to real world settings (the efficacy-effectiveness gap). In practice, then, there will at least sometimes (and perhaps often) be scope for reasonable disagreement among experts about whether the data to hand are sufficient for the ethically acceptable use of the intervention in specific real-world populations. Taking this disagreement seriously could not only contribute to trust in experts, but also potentially maximize the benefits of vaccine research. Failures to heed the views of the reasonably minority of experts that were uncertain about the net medical benefits of COVID-19 vaccines in specific groups, such as the JCVI, arguably foreclosed opportunities to collect more data, including regarding alternative dosing strategies for mRNA vaccines (Prasad 2023) and long-term outcomes from myocarditis (Kracalik et al. 2022). Consequently, it resulted in authorization of COVID-19 vaccines even for groups where there may have been net expected harm (Bardosh et al. 2022).

Being transparent about these types of disagreements may allow policymakers to authorize the use of vaccines and strongly recommend them for high-risk groups while (in the face of uncertainty) refraining from strong recommendations or mandating vaccination in low-risk groups.

## 5 Trust’s Role in Public Health and Covid-19

New vaccines might be authorized and recommended at the population level based on a majority expert consensus regarding net benefit, but where a reasonable minority of experts disagree (Figure 2B). If the concerns of the minority turn out to be well founded but ignored, this can lead to preventable harms unforeseen by the majority. For example, in 2015-2017, a vaccine for dengue was approved for use and implemented in some countries despite at least one group of experts having expressed concerns about potential risks (Jamrozik et al. 2021). When the harm foreseen by this group of experts eventuated, public trust in vaccination more generally plummeted in relevant communities. This increased mistrust was considered by some authors “a threat to pandemic preparedness” (Larson et al. 2019). Below, we examine how trust in experts could be undermined by decisions about vaccine policy, especially when they concern a novel vaccine during a pandemic.

### 5.1 Case Study: COVID-19 Vaccine Mandates and Trust

The COVID-19 pandemic has been characterized by some lack of clarity surrounding public health agencies’ decision-making processes, recommendations, and policies, including on social media (Reveilhac 2022)

In implementing vaccine policy, policymakers have limited public trust with which to barter. Moreover, restrictive policies are likely to undermine trust (Nature 2018), as the fall in confidence in science during a pandemic characterized by restrictive response measures might suggest. Indeed, COVID-19 vaccines were also mandated for low-risk groups in some settings, such as many North American universities (Bardosh et al. 2022). Vaccine mandates might be effective at increasing vaccine uptake in the short term. However, even when they are, if they produce a decrease in trust in public health and in vaccine experts, they can undermine vaccine uptake in the long term (Abrevaya and Mulligan 2011). This might ultimately outweigh the potential benefit rendered from an increase in vaccine uptake (Bardosh et al. 2022).

Ethical analysis of COVID-19 mandates that took into consideration the benefits of a rise in vaccination rates against potential negative risks, such a loss of healthcare workforce and trust in vaccines due to vaccine mandates, was largely missing from public debate before their implementation (Gur-Arie et al. 2021). Meanwhile, COVID-19 vaccine mandates were sometimes sold as a “band aid” solution to systematic public health challenges, such as insufficient hospital capacity. The ongoing evolution of COVID-19 vaccine mandates and policy is an interesting case in understanding how fast-paced, “well-intentioned” policymaking during a pandemic without thorough and transparent ethical analysis can, ultimately, backfire in terms of public trust (Holton 2021).

### 6.2 Case study: COVID-19 Vaccine Policy for Children

Infant and child vaccination has not always been easily accepted and implemented across society. Primary drivers of modern parental vaccine hesitancy towards child vaccination include religious reasons, personal beliefs or philosophical reasons, and safety concerns (McKee and Bohannon, 2016). Some communities of parents have always been, and probably will continue to remain, hesitant towards some or all vaccines for their children (Bernstein et al, 2019).

The COVID-19 pandemic exacerbated mistrust in vaccines (He et al, 2021). Routine childhood vaccination rates have declined compared to pre-pandemic levels (Balch 20222; Bramer et al., 2020; Nolen 2022). Regarding COVID-19 vaccines, as of early 2023 only 10% of parents in the US and Canada have chosen to vaccinate their children under 5 years old. In both countries, less than 50% of children aged 5-11 have received 2 doses (AAP 2023, Government of Canada 2023). In the UK, 9 out 10 children aged 5-11 are not vaccinated against COVID-19 (ONS 2023). All this happens while policy and presumably the majority view among experts is that such vaccines are medically beneficial for children. This situation seems to suggest widespread lack of trust in vaccine and public health experts and/or uncertainty about net benefits in this group. Given our arguments above, there is the risk that relevant experts could lose some of their authority in the eyes of most parents.

The process of rebuilding or reinforcing trust should be sensitive to many legitimate concerns surrounding vaccine development and appropriate safety data, such as concerns related to Pfizer’s failure to follow FDA’s instructions to conduct further studies into mRNA vaccine myocarditis (Prasad 2023). As argued above, trustworthiness also involves being transparent about uncertainty and disagreement. It requires acknowledging that the minority view of experts who recommended against using COVID-19 vaccines for certain low risk groups might, after all, be right, or at least worth listening to.

Decision-making surrounding vaccine policy has the potential to affect public trust in vaccines more generally. This might also have broader implications for trust in scientific expertise, institutions, and public health agencies. COVID-19 vaccine hesitancy is impacted by many factors that usually influence vaccine hesitancyn– confidence, complacency, convenience, communication, and context (Razai et al. 2021). Hesitancy that arose during the COVID-19 pandemic was particularly unique in that the timelines for vaccine development were especially rapid, levels of global public scrutiny were high, and, in some countries, punitive measures for unvaccinated people were severe (Liebermann and Kaufman, 2021).

Despite the celebrated pace of developing and rolling out COVID-19 vaccines in one year, the “warp speed” of the operation has also been met with expert and public concern surrounding “how fast” is “too fast” (Van Norman 2020). Children were left out of initial safety trials for COVID-19 vaccines. As a result, COVID-19 vaccine rollout in children began around one year later than in adults. As we saw, whether mRNA COVID-19 vaccines offered net benefit to children was, at the time, not a consensus among the scientific community, or even among public health authorities. At least some concerns surrounding the safety of the vaccine for certain pediatric populations, like adolescent boys, emerged *after* cases of myocarditis post-vaccination (Vogel and Couzin-Frankel, 2021).

Given these uncertainties, it is unsurprising that trust in vaccine and public health experts decreased, affecting childhood vaccination generally -- even impacting vaccines that previously enjoyed quite widespread parental support.

## 7 Conclusions

Trust is cornerstone to the success of public health interventions like vaccines – particularly infant and child vaccination. Decreased levels of trust in vaccines, experts, and public health agencies has already led to decreased global levels of vaccine uptake, particularly among children (Larson et al. 2022). Whom we take to be vaccine experts can be affected by the way expert disagreement and uncertainty are acknowledged both by experts themselves and by public health authorities. This is because attributions of expertise are acts of trust in epistemic but also in moral features of the person we, as individuals and as society, decide to consider experts. The epistemic and the moral dimensions of trust are inseparable from each other because satisfying epistemic conditions requires committing to some ethical principles. These include humility, honesty, and transparency regarding the limitations of one’s knowledge, which would prevent epistemic failures such as overconfidence. To the extent that it contributes to preserving public trust, transparency about expert disagreement and uncertainty is an essential aspect of what it means to be an expert. Indeed, it can as important as being confident in what one, as an expert, believes to be true.

## Footnotes

1 See e.g. L Fisher, L Robert and L Turner 2021, Get your children vaccinated against Covid, parents are told, *the Telegraph*, 13 Sept 2021, at https://www.telegraph.co.uk/news/2021/09/13/chief-medical-officers-approve-vaccinesteenagers/), accessed 9 January 2023. It is worth noting that this line of argument can be questioned, see e.g Alberto Giubilini, The Double Ethical Mistake of Vaccinating Children against COVID-19, *Practical Ethics. Ethics in the News* blog, 13 September 2021, at https://blog.practicalethics.ox.ac.uk/2021/09/the-double-ethical-mistake-of-vaccinating-children-against-covid-19/, accessed 12 January 2023

2 We thank an anonymous reviewer for this observation

3 Berlkey Lovelace and Tyrrell, Two senior FDA vaccine regulators are stepping down, *CNBC* 31 August 2022, at https://www.cnbc.com/2021/08/31/two-senior-fda-vaccine-regulators-are-stepping-down.html, accessed 12 Jan 2023,
